# Modulating the Crosstalk between the Tumor and the Microenvironment Using SiRNA: A Flexible Strategy for Breast Cancer Treatment

**DOI:** 10.3390/cancers12123744

**Published:** 2020-12-13

**Authors:** Giuseppina Roscigno, Iolanda Scognamiglio, Francesco Ingenito, Rosario Vincenzo Chianese, Francesco Palma, Alan Chan, Gerolama Condorelli

**Affiliations:** 1 Percuros BV, Albinusdreef 2, 2333 ZA Leiden, The Netherlands; g.roscigno@percuros.nl (G.R.); francesco.ingenito@outlook.it (F.I.); frapalma9@gmail.com (F.P.); achan@percuros.com (A.C.); 2 Department of Molecular Medicine and Medical Biotechnology, “Federico II” University of Naples, Via Pansini 5, 80131 Naples, Italy; iolanda.scognamiglio@unina.it (I.S.); rosario.chianese96@gmail.com (R.V.C.); 3 Istituto per l’Endocrinologia e l’Oncologia Sperimentale “G. Salvatore” (IEOS), Consiglio Nazionale delle Ricerche (CNR), 80131 Naples, Italy

**Keywords:** breast cancer, siRNA, TME, cancer therapy

## Abstract

**Simple Summary:**

With this review we aimed to collect the most relevant scientific findings regarding siRNA therapeutic tools against breast cancer microenvironment. Remarkably, breast cancer treatments have been redirected towards the tumor microenvironment components, mainly involved in patients’ relapse and pharmacological resistance. Therefore, siRNAs represent a promising strategy to jeopardize the tumor microenvironment interplay thanks to their non-toxic and specific effects.

**Abstract:**

Tumorigenesis is a complex and multistep process in which sequential mutations in oncogenes and tumor-suppressor genes result in enhanced proliferation and apoptosis escape. Over the past decades, several studies have provided evidence that tumors are more than merely a mass of malignant cancer cells, with the tumor microenvironment (TME) also contributing to cancer progression. For this reason, the focus of cancer research in recent years has shifted from the malignant cancer cell itself to the TME and its interactions. Since the TME actively participates in tumor progression, therapeutic strategies targeting it have created great interest. In this context, much attention has been paid to the potential application of small interfering RNA (siRNA), a class of non-coding RNA that has the ability to downregulate the expression of target genes in a sequence-specific way. This is paving the way for a novel therapeutic approach for the treatment of several diseases, including cancer. In this review, we describe recent efforts in developing siRNA therapeutics for the treatment of breast cancer, with particular emphasis on TME regulation. We focus on studies that adapt siRNA design to reprogram/re-educate the TME and eradicate the interplay between cancer cells and TME.

## 1. Introduction

Breast cancer is the most frequently diagnosed cancer in women and, despite the important improvements in prognosis and in therapy that have been achieved in recent decades, remains the first cause of cancer-related death in women worldwide. It encompasses a collection of distinct phenotypes, which can be categorized in three major clinical subtypes: estrogen receptor (ER)/progesterone receptor (PR)-positive, human epidermal growth factor receptor type 2 (HER2)-positive, and triple-negative (ER−/PR−HER2−) breast cancers.

ER/PR+ cancer accounts for 60–70% of all breast cancers [1,2]. This subtype has the best prognosis [3], with median survival of about 60 months. The first-line treatment is endocrine therapy based on specific estrogen receptor inhibitors such as fulvestrant or aromatase inhibitors [2]. However, resistance often arises, and several clinical trials have evaluated new drugs, such as inhibitors of CDK4/6 and immune checkpoints, in combination with endocrine therapy [4].

The HER2+ subtype accounts for about 15–25% of invasive breast cancer [5,6] and is characterized by a poor prognosis [7]. The administration of the monoclonal antibody Trastuzumab (the first HER2-targeted therapy) has substantially improved the prognosis of this subtype [8], but resistance to treatment is still common. Actually, there are newer HER2-targeted therapies, such as pertuzumab, lapatinib, and neratinib, that have been used with trastuzumab [9,10,11] to overcome resistance, with the best results achieved with a combination of pertuzumab, trastuzumab, and taxane [12]. Thus, this combination is used as the first-line treatment for HER2+ breast cancer, improving progression-free survival to 18.5 months compared to 12.4 months on trastuzumab + docetaxel [12].

Triple-negative (TNBC, ER−/PR−/HER2−) cancer represents about 15% of all breast cancer. Treatment for TNBC is based on new chemotherapeutic drugs (i.e., not previously used) or a different combination of drugs (e.g., doxorubicin + paclitaxel). Unfortunately, when also considering these treatments, median overall survival from metastasis to death is about 11 months for TNBC or up to 2.2 years in the case of less aggressive types [13]. Most recently, the combination of atezolizumab, a Programmed Death (PD)-Ligand 1 immune checkpoint inhibitor, with nab-paclitaxel was approved for patients with metastatic TNBCs that overexpress PD-L1 [14]. However, progression-free survival in TNBC patients with PD-L1-positive expression and subjected to atezolizumab/nab-paclitaxel treatment is only 7.5 months, compared to 5.0 months with nab-paclitaxel alone [15]. These insufficient effects of treatments require the development of new approaches to address urgent medical needs.

## 2. Tumor Microenvironment (TME) in Breast Cancer

The TME is a crucial player in the progression of tumors, including breast cancer. Indeed, cancer cells can transform fibroblasts, immune cells, and other resident cells into tumor-promoting cells (activated cells), which in turn secrete growth factors, cytokines, intermediate metabolites, as well as matrix components that remodel the TME. All of these mediate a crosstalk with the tumor that facilitates progression and sustained adaptation according to the tumor status.

### 2.1. Cancer-Associated Fibroblasts (CAFs)

CAFs are the main component of breast cancer stroma [16]. During tumor progression, fibroblasts are transformed into activated fibroblasts called CAFs thanks to Transforming Growth Factor β1 (TGFβ1) [17], Wnt7a, and other factors secreted by the tumor cells [18,19]. CAFs are involved in proliferation, angiogenesis, and invasiveness and orchestrate the metastatic process in at least two different ways: first, they induce EMT through activation of TGF-β receptor signaling and extracellular matrix (ECM) remodeling [20]; second, CAFs release inflammatory interleukins, non-coding RNAs [21], interferons, and tumor necrosis factor α (TNFα) in tumor sites, contributing to the accumulation of innate and specific immune cells, such as macrophages, and thus induce their activation. Moreover, CAFs recruit regulatory T cells (Tregs) through the secretion of various chemokines, like CCL5 [22]. These activated immune cells, in turn, promote the metastatic potential of cancer cells.

### 2.2. Vasculature

The vasculature is a key element in cancer progression. It consists of an inner layer of endothelial cells enveloped by pericytes, responsible for vessel integrity. In larger vessels, an outer layer of smooth muscle cells is also present. Tumoral and stromal cells release Vascular Endothelial Growth Factor (VEGF), inducing the activation of normal quiescent vasculature and promoting the sprouting of new vessels [23,24]. In this manner, new vessels provide nutrients and oxygen for tumor growth and escape.

### 2.3. The ECM

The ECM is the non-cellular component of the TME. It is composed of water, fibers, and proteins, and has mechanical and physiological functions promoting cell communication, adhesion, proliferation, and metastasis [25]. The ECM is composed of different fibrin proteins, such as laminin, elastin, proteoglycan, and collagen. For breast cancer, the stromal microenvironment appears stiffer than normal due to increase deposition of collagen I, II, III, V, and IX [26] and overproduction of heparin sulphate proteoglycans [27]. Significantly, increased collagen abundance and reorganization has been associated with breast cancer progression [28,29,30]. Another important component of the ECM is glycosaminoglycan. Glycosaminoglycans (GAGs) are highly sulfated, negatively charged polysaccharide chains that mediate a wide variety of biological functions. These components are often used to propagate signals in development, cell adhesion, immunity, and cell replication [31,32] [33], and their alteration is also involved in cancer [34,35,36,37].

### 2.4. Immune Cells and Dendritic Cells (DCs)

CD4+ and CD8+ T cells belong to the acquired immunity arm and are involved in immune surveillance. CD8+ T lymphocytes differentiate into Cytotoxic T lymphocytes (CTLs), which are the major effector cells against breast cancer [38]. CTLs recognize tumoral antigens exposed on MHC class 1 of the tumor cells, and then release perforin and granzymes, which kill cancer cells [39]. Elevated levels of cytotoxic CD8+ T cells in the TME have been linked to positive anti-tumor effects in breast cancer [40]. CD4+ T cells can differentiate into different effector subtypes, the most common being T helper-1, -2, and -17 (Th1, Th2, and Th17) cells and regulatory T (Treg) cells [41]. Th1 cells secrete interleukin (IL)2, IFN-γ, and TNF-α, which stimulate macrophage antitumor activities. On the contrary, Th2 cells secrete pro-tumoral cytokines, such as IL-4, IL-5, IL-6, IL-10, and IL-13, which induce anergy in T cells and promote the activities of tumor-promoting macrophages [41]. Tregs have been reported to promote tumor progression in breast cancer by suppressing the activities of CTLs and Th1 cells [42].

Tumor-associated macrophages (TAMs) belong to the innate immunity arm. In breast cancer, TAMs can constitute over 50% of the number of cells within the tumoral mass [43]. TAMs are divided into two categories: M1 and M2. M1 macrophages have a pro-inflammatory function and an antitumoral activity [44,45] through the secretion of pro-inflammatory cytokines, such as TNF and interleukin (IL)-2, and the stimulation of CTL response against cancer cells. In contrast, M2 macrophages create a pro-tumoral microenvironment [45] by secreting anti-inflammatory molecules (e.g., CCL18, IL10, growth factor, cytokines), promoting invasion through the secretion of metalloproteinases (MMPs) and inhibiting infiltration and function of antitumor CTLs. Most TAMs in the tumor microenvironment are closely related to the M2 phenotype, and their infiltration level has been linked to poor prognosis in breast cancer, higher-grade tumors, and worse overall survival [46].

DCs serve as linkers between innate and adaptive immunity [47] and are crucial regulators for T-cell-mediated cancer immunity. After antigen internalization [48], DCs transport tumor antigens to draining lymph nodes and present antigen to CD8+ T cells. DC maturation is necessary to provide costimulatory signals to T cells, inducing their activation into CTLs, but DC maturation is often insufficient to induce potent immunity, particularly when a suppressive microenvironment is preset within tumors. Since DCs play a critical role in promoting anti-tumor T cell immunity, they represent a major therapeutic target.

### 2.5. Adipocytes

Another crucial cell type in breast cancer TME is the adipocyte [49]. Adipocytes communicate with surrounding cells by secreting pro-inflammatory cytokines (IL-6, IL-1β, and TNF-α) and MMPs, which modulate cancer metabolism. In particular, interleukin-6 (IL-6) induces tumor cell invasiveness and increases the metastatic ability of tumor cells [50]. The secretion of TNFα by adipocytes increased metastasis in co-culture experiments and animal models [51,52]. In addition, adipocytes promote tumor progression through the secretion of various hormones, such as estradiol (E2), prolactin, leptin, and adiponectin, which activate proliferation and survival pathways. Altogether, this crosstalk promotes the development of a hostile TME that is reflected in the poor clinical outcome of breast cancer patients [50].

## 3. siRNA and Clinical Applications

RNAi is a post-transcriptional gene silencing mechanism participating in the natural process of resistance to the invasion of pathogenic, exogenous, double-stranded RNA; it is executed by three types of small non-coding RNAs (microRNA, siRNA, and short hairpin RNA (shRNA)) [53,54]. siRNA and miRNA can knock down the expression of target genes in a sequence-specific way by inducing mRNA degradation (for siRNA and miRNA) or blocking mRNA translation (for miRNA), a kind of post-transcriptional regulation widely investigated in cancer [55,56,57]. siRNA consists in a double strand of RNA containing a homologous sequence to a specific gene, with the ability to silence it. The process starts in the cytoplasm, where the enzyme Dicer cleaves a double-stranded RNA into a shorter double strand 21–25 nt (siRNA) in length. The guide strand is loaded into the RNA-induced silencing (RISC) complex, and when it recognizes, via a perfect match, a target mRNA, the RISC complex leads to the cleavage of the mRNA. In this manner, siRNA can regulate gene expression, triggering efficient and specific gene silencing. By mimicking this natural process, it is possible to control the expression of specific disease genes, leading to precise and personalized treatments. Several in vitro studies have shown the efficiency of this mechanism in numerous pathologies, including cancer [58].

From a therapeutic point of view, siRNAs have several advantages with respect to small therapeutics drugs and monoclonal antibodies. Indeed, they act through perfect base pairing with mRNA, whereas small molecules and monoclonal antibodies need to recognize the complex tridimensional conformation of a target protein. Furthermore, any gene of interest can be easily targeted by an siRNA, since only a complementary nucleotide sequence on the targeted mRNA is needed to be designed, whereas conventional compounds can target only a subset of proteins/pathways, defined as “druggable” targets.

Although siRNAs are promising, several barriers limit clinical application. Unmodified siRNAs have some disadvantages, such as inadequate stability in the circulation due to the presence of RNAses that may quickly degrade them, a poor pharmacokinetic properties, and the possible induction of off-target effects if an imperfect match is tolerated by the RISC, leading to undesirable silencing of genes [59]. Moreover, siRNA can trigger the activation of Toll-like receptor 3 (TLR3), negatively influencing the immune system. The occurrence of side effects of siRNA has thus raised many doubts on their possible clinical use. Indeed, siRNA-based therapeutics has suffered many ups and downs since the discovery of siRNA in 1998. To maximize treatment efficacy and reduce the side effects of siRNA, researchers have focused much effort in recent years on the identification of chemical modifications that would aid the development of delivery systems [60].

Interestingly, Patisiran has been recently approved by FDA as the first siRNA therapeutic for the treatment of hereditary amyloidogenic transthyretin amyloidosis. Givosiran has also been recently approved by the FDA for the treatment of acute hepatic porphyria [61,62,63,64]. Thus, siRNA technology may also be promising for the treatment of several other diseases, such as cancer. There are no RNAi-based drugs approved for anticancer therapy, but several therapeutics are currently undergoing clinical trials. Clinical trial involving Ephrin type-A receptor 2 (EphA2) and M2 subunit of ribonucleotide reductase (R2) siRNA-mediated therapy are currently ongoing for Hepatocellular carcinoma (HCC)patients and solid tumors, respectively [65,66,67].

## 4. siRNA Therapeutics Targeting the TME

siRNA can have anticancer effects thanks to an ability to silence overexpressed genes involved in cell proliferation, drug resistance, and metastasis. RNAi could be used also to modify the secretory profile of other cells and interrupt crosstalk between the tumor and the surrounding microenvironment, an essential component of tumor progression. In the following sections, we will describe recent work on siRNA technology as a promising and fascinating therapeutic tool against the breast cancer cell–TME network summarized in Table 1.

### 4.1. siRNAs against CAFs

CAFs represent the majority of the cells composing the tumor mass. They can promote tumor progression through the release of pro-angiogenic factors and mediators involved in the inflammatory pathway [78,79]. In this scenario, an interesting study of Liubomirski et al. [80] demonstrated the prominent role of tumor stroma inflammation networks in promoting the aggressiveness of TNBC. In particular, siRNA-mediated silencing of C-X-C Motif Chemokine Ligand (CXCL) 8 in breast cancer and stromal cells significantly decreased endothelial cell migration and partly reversed the relevant morphology of the tumor cells, so leading to a reduction of tumor migration and invasion potential. 

CAF activation and maintenance depends also on the activation of Yes-Associated Protein 1 (YAP1) transcriptional programs, which regulate the contractile actomyosin cytoskeleton. Interestingly, Calvo et al. [68] found YAP1 to be more expressed and nuclearly localized in CAFs compared to normal and hyperplasia-associated fibroblasts, both in mice and in human breast cancer biopsies. Notably, when YAP was silenced by siRNAs, the ability of CAFs to contract the collagen matrices was strongly reduced, as were the formation of focal adhesions, the generation of collagen fibers, and angiogenesis in vivo. Moreover, Yan-e Du et al. [81] demonstrated how the miR-205/YAP1 axis in CAFs can promote angiogenesis by regulating IL-11 and IL-15 levels, which in turn up-regulate the STAT3 protein level in endothelial cells. Consequently, IL-11 and IL-15 knock-down mediated by specific siRNAs in CAFs resulted in the impairment of angiogenic ability in Human Umbilical Vein Endothelial Cells (HUVECs), with a final silencing efficiency comparable to that obtained by anti-IL-11- and/or anti-IL-15- specific antibodies.

A recent paper identifies fibroblast-expressed PIK3Cδ as new mediator for TNBC progression. The authors used an interesting approach consisting in the silencing of 710 kinases in fibroblasts through a siRNA kinome library. Then, fibroblasts were cocultured with MDA-MB-231 cells, and the formed spheroids were plated in Matrigel in order to promote invasion. The authors found that this PIK3Cδ kinase modulated MDA-MB-231 invasion in a paracrine way and that its inhibition could represent an alternative therapeutic option for TNBC treatment [82].

Given the high specificity of siRNAs in downregulating stroma-related pathways and the role of CAFs in tumor recurrence, metastasis formation, and chemoresistance [83], the use of fibroblast-directed siRNAs in combination with canonical therapeutic drugs could represent a turning point in breast cancer treatment. Indeed, this strategy has already been tested in ovarian cancer to overcome chemoresistance. In particular, siRNA-mediated silencing of lipoma preferred partner (LPP) in CAFs improved the delivery of cytotoxic drugs to ovarian cancer cells in vivo [84]. Moreover, because it is also involved in breast cancer invasion and migration [85], LPP protein could be investigated as a putative target for innovative combinatorial treatments targeting breast TME.

### 4.2. siRNAs Targeting Angiogenesis

The pro-angiogenic role of the TME has been extensively investigated. Crosstalk between the TME and cells has been associated with the promotion of tumor angiogenesis [86], suggesting putative candidates for therapeutic ends.

The canonical pro-angiogenic pathways have been widely explored, and antiangiogenic drugs against VEGF or VEGF receptor (VEGFR) (including the monoclonal antibodies Bevacizumab, Axitinib, Sorafenib, and Sunitinib) have been developed and approved. However, in most treated patients these therapies have failed because of multiple drug-resistance mechanisms [69]. Therefore, there is a need for new therapeutic strategies to target angiogenesis.

In this context, delivery of siRNA specifically into endothelial cells is a promising strategy to impair tumor angiogenesis. To this end, a study by Egorova et al. [69,70] showed how siRNA-mediated silencing of vascular epithelial growth factor signaling (VEGF-A, VEGFR1) and endoglin, a co-receptor for TGF-β, impaired endothelial cell migration and proliferation. However, an efficient and specific siRNA delivery system is crucial for the development of this promising approach. In this regard, they used a modular L1 peptide carrier containing a ligand for the CXC4 receptor (CXCR4), which is widely and specifically expressed on the endothelial cell surface.

Similarly, an alternative approach to target cancer angiogenesis using an siRNA-based silencing approach was reported by Bharti and colleagues, who focused on IL-6/IL-6R and mitochondrial signaling in breast cancer. The authors showed how the inhibition of IL-6/IL-6R signaling by IL-6 siRNA suppressed angiogenesis/invasion by up-regulating MAO-A expression, a mitochondrial protein that commonly degrades monoamines and is usually found down-regulated in multiple cancers, including breast carcinoma [87,88].

Finally, an interesting paper demonstrated how senescence of HUVEC cells induced by ionizing radiation and doxorubicin treatment increased MDA-MB-231 cancer cell proliferation and invasion through (CXCL11) secretion. Furthermore, when the authors treated HUVEC with a CXCL11 siRNA, or treated MDA-MB-231 cells with a siRNA for CXCR3 receptor, they observed a strong inhibition of the ability of the senescent HUVEC to alter the spheroid invasion of cancer cells, demonstrating that CXCL11 can be targeted to hamper the adverse effects of therapy induced-senescent endothelial cells [89].

### 4.3. siRNA Targeting ECM

TME-mediated cell migration is the key process in metastasis formation and correlates with poor prognosis in patients [90]. Therefore, understanding the molecular machinery that orchestrates the continuous remodeling of the cytoskeleton and cell–matrix adhesions is a fundamental starting point for the identification of new drug targets against cancer progression. An exemplary study carried out by Fokkelman and colleagues [91] identified myosin phosphatase target 2 (PPP1R12B), Homeodomain Interacting Protein Kinase 3 (HIPK3), and Ras-related C3 botulinum toxin substrate 2 (RAC2) as the main modulators of cellular traction forces and cell migration mechanisms in breast cancer. Interestingly, the siRNA-mediated down-regulation of these three proteins in the MCF-7 breast cancer cell line resulted in the impairment of cellular migration and in a sustained increase of force application in vitro. Furthermore, the effect of siRNA-mediated knock-down of PPP1R12B, RAC2, or HIPK3 strongly attenuated the dynamics of focal adhesion, leading to increased cell connections to the ECM.

siRNA-mediated silencing of β4GalT7 and EXT1, the central glycosaminoglycan (GAG) synthetic enzymes that modulate numerous cellular processes relevant to tumor progression, including cell proliferation, cell-matrix interactions, cell motility, and invasive growth, is another novel and interesting approach [92,93]. Here [93], the downregulation of β4GalT7 decreased migration and proliferation of MDA-MB-231 cells but, unexpectedly, increased adhesion to fibronectin (FN). Despite cellular migration and proliferation features being impaired by this anti-tumorigenic approach, increased adhesion to FN negatively correlated with anchorage to the ECM. For this reason, as observed by the authors, the abrogation of either β4GalT7 or EXT1 enzymes has helpful, but inconclusive, effects in decreasing cancer cell aggressiveness. This highlights the need to carefully investigate any multi-targeted approach. Nevertheless, the efficacy of a combinatorial siRNA strategy against multiple targets is a promising therapeutic approach, especially for breast cancer, in which the targeting of TME components along with cancerous cells is needed.

### 4.4. siRNA Targeting TAMs

TAMs play a major role in breast TME [94], and several papers have reported that breast cancer cells promote the recruitment of new monocytes, inducing their differentiation from an M1 anti-tumorigenic phenotype into pro-tumoral M2-TAMs [95]. Numerous approaches have been developed to directly target TAMs and repolarize them to the M1 phenotype. Notably, TAMs have been reported to sustain tumor angiogenesis through the production of vascular endothelial growth factor (VEGF) in several types of cancer (e.g., pancreatic and gastric cancer, lung adenocarcinoma, and melanoma) [96]. Although different classes of molecules (e.g., monoclonal antibodies and multiples tyrosine kinase inhibitors) have been commonly used in clinics over the past 10 years, resistance to anti-angiogenic therapies still represents an issue in oncology [97]. Therefore, siRNA may act as an accurate and nontoxic tool to overcome such problems or to synergize the effect of antiangiogenic therapy. In 2018, Song et al. [97] elaborated a dual-siRNA strategy targeting VEGF and its analogue, Placental Growth Factor (PIGF), which is usually over-expressed in M2-TAMs. These two factors play a crucial role in tumor growth, since it was demonstrated that their release in the microenvironment promotes angiogenesis and hypoxia. Interestingly, the synergistic effect of VEGF and PIGF silencing is also able to revert the phenotype from M2 to M1, as confirmed by in vitro and in vivo downregulation of IL-10 and CTLA-4 and upregulation of IL-6 and IFN-γ, a molecular signature indicating a less immunosuppressive microenvironment. Therefore, as proven by Song et al., combined inhibition of VEGF and PIGF impacts the whole TME by reducing breast cancer cell growth and angiogenesis [71].

Several pathways have been linked to M2 activation. Indeed, upon stimulation of tumor-secreted IL-4, IL-10, and IL-13, macrophages can be directed towards an M2 phenotype with up-regulation of crucial pathways such as Signal Transducer and Activator of Transcription (STAT)3 and STAT6 [75]. Even though STAT family proteins are well known to be linked to the aggressiveness of several solid (e.g., breast, prostate, thyroid, and lung) and hematopoietic tumors, they remain difficult to target [98]. Despite the approval of STAT pathway inhibitors, such as Ruxolitinib or Tofacitinib, by the FDA, the development of specific STAT-targeting drugs is still challenging, especially due to the high level of homology within the STAT protein family [99]. Given their specificity and lack of toxicity and immunogenicity, siRNAs are promising tools to specifically target one or more members of the STAT family with single- or multi-siRNA approaches. In this regard, Binnemars-Postma et al. [72] came up with an siRNA to specifically inhibit STAT6 signaling in vitro in M2-TAMs, obtaining conversion to the M1 phenotype. Additionally, the authors examined in vivo the activity of a STAT6 pharmacological inhibitor. Although this molecule hampered M2 pro-tumorigenic effects and reduced ECM remodeling and metastasis, it presented with considerable toxicity. Thus, STAT6 siRNA may be a good candidate to overcome such adverse effects or as an adjuvant to pharmacologic therapy.

A different approach to impair M2 functions is to hamper the complex tumor–stroma interplay, monocyte/macrophages recruitment, and subsequently M2 switch. In this context, Yang et al. [100] identified EGF as a key growth factor that stimulates, in a paracrine manner, M2-TAM activity in xenograft mice. Firstly, they found that upon EGF stimulation, the STAT3/Sox-2 pathway is strongly upregulated in breast cancer cells, heading to uncontrolled cell proliferation, chemoresistance, maintenance of cancer cell stemness, and metastasis. Specific STAT3 siRNA-mediated inhibition in neoplastic cells may successfully suppress M2-TAM pro-tumorigenic activity and the downstream biological effects on the TME. Therefore, siRNA-mediated targeting of immune infiltrates is a promising, novel, specific, and non-toxic approach for breast cancer treatment.

### 4.5. siRNA DCs

One of the most modern immunotherapy-based tools is DC vaccine: ex vivo antigen-mediated pulsed DCs are reinfused into the patient to induce a massive anti-tumoral T-cytotoxic response [101]. In the context of the TME, DCs contribute to endorse a response against the tumor [102]. However, dysregulation of several molecular markers, such as the tryptophan-degrading enzyme named Indoleamine 2,3-DiOxygenase (IDO), is associated with alteration of DC function [103]. Indeed, IDO overexpression in DCs is associated with a poor T-cytotoxic response against the tumor, since T cells are highly sensitive to tryptophan concentration, and its degradation products induce T-cell apoptosis [103]. Zheng et al. [76] engineered a novel DC vaccine using mouse DCs that were first treated with an IDO siRNA and then pulsed with 4T1 breast cancer cell line lysate. The administration of the vaccine in 4T1-harboring mice decreased CD4+ T cells and Treg cells within the TME, resulting in an enhanced antitumoral effect.

Interestingly, Hira et al. [104] found that DCs have a direct and proactive role against neoplastic cells. Co-culture of breast cancer cells (MDA-MB-415 and MCF-7 cells) with derived primary DCs reduced levels of CD24 [105] and HER-2 [106], two important proteins in breast cancer proliferation and metastasis. When the authors silenced STAT3 in breast cancer cells and co-cultured them with fresh blood-derived DCs, they found that the treatment strongly synergized CD24 and HER2 downregulation in the cancerous cells.

### 4.6. siRNA Targeting Immune Infiltrates

Treg cells are a promising target for modern immunotherapy approaches. Indeed, several FDA-approved compounds, including monoclonal antibodies or low doses of cytotoxic drugs, have been recently used to jeopardize the pro-tumorigenic function of Treg cells [107]. However, these approaches cannot always be applied to clinical practice [108,109], leaving a hole that might be filled by siRNAs.

Su et al. [110] focused on PYK2 N-Terminal Domain-Interacting Receptor (PITPNM) 3, a CD4+ T cell surface receptor that drives intra-tumoral Treg recruitment. They engineered an aptamer–siRNA chimera to improve the delivery of PITPNM3 siRNA to CD4+ T cells in a humanized mouse model of breast cancer. Intraperitoneal administration of this chimera resulted in a reduction in Treg recruitment and an increase in the CD8+ T cell population, promoting apoptosis, reducing tumor growth, and blunting lung metastases.

Another modern and promising approach in oncology is represented by immune checkpoint inhibitors (ICIs). The blockade of negative regulators of the immune response, such as PD-1/PD-L1 and CTLA-4 axes, using monoclonal antibodies boosts the immune system response against neoplastic cells [77].

Although targeting the PD-1/PD-L1 pathway is currently a common clinical practice, not all TNBC patients respond to this kind of therapy [111]. Therefore, siRNAs may help to develop new personalized therapies in the context of PD-1/PD-L1+ tumors and, eventually, to overcome ICI resistance and severe side effects. For this purpose, Li et al. engineered a nano-delivery system based on infiltrating peptides that simultaneously carry PD-L1 siRNA and 1-MT, a pharmacological inhibitor of IDO enzyme. In vitro and in vivo studies proved that downstream effects of PD-L1 knockdown were improved by 1-MT. Indeed, the synergistic effect of these two molecules reduced tumor growth in 4T1 breast cancer-bearing mice [73].

Neoplastic cells undergo deep metabolic changes, dysregulating various pathways to sustain abnormal tumor growth and proliferation [112]. Cancer metabolism affects the whole TME and drives the insurgence of drug resistance, jeopardizing immunotherapy efficiency [113]. El Ansari and colleagues [114] developed an siRNA-based strategy targeting SLC7A5, which mediates the efflux of glutamine and the influx of leucine and is upregulated in breast cancer [115]. Interestingly, siRNA-mediated SLC7A5 knockdown resulted in a decrease in PD-L1 expression level within the tumor.

### 4.7. siRNA Targeting Cancer-Associated Adipocytes

Lehuédé and colleagues investigated the role of mammary adipose tissue in drug resistance in breast cancer cells [116]. One of the main mechanisms underlying multi-drug resistance (MDR) in breast cancer is the upregulation of ABC family transporters, which mediate drug extrusion from neoplastic cells [117]. Major Vault Protein (MVP) is a main player in MDR, becoming upregulated in several cancer cell lines [118]. Lehuédé et al. [116] demonstrated how in vitro co-cultures of human and murine breast cancer cell lines with adipocytes strongly upregulates tumoral MVP expression, inducing MDR. Consequently, silencing MVP with an siRNA-mediated approach results in massive accumulation of antineoplastic compounds (e.g., doxorubicin and 5-FU) within cancerous cells, suggesting siRNA as a valid alternative strategy to overcome MDR.

Among the several soluble factors that mediate the complex interplay between cancer-associated adipocytes and neoplastic cells, leptin plays a crucial role, since it is one of the major adipocyte-secreted hormones [119]. Wei et al. discovered how adipocyte-derived leptin elicits several biological effects in neoplastic leptin receptor-overexpressing cells (e.g., MDA-MB-468, MCF-7, and SK-BR-3 cells). In such cell lines, leptin induced EMT via downregulation of E-cadherin and strong upregulation of Vimentin and Fibronectin [74]. Wei et al. [120] found that Pyruvate Kinase M2 (PKM2), a protein commonly known for its role in aerobic metabolism and strongly upregulated in cancer cells, also works as a transcription factor. Therefore, siRNA-mediated PKM2 silencing results in inhibition of leptin-induced cell invasion and migration in vitro and a decrease of all EMT-related markers. Furthermore, since PKM2 enhances PI3K/AKT signaling, the authors tested several pharmacological compounds to block this pathway [74]. Consequently, siRNA may be useful in multidrug therapy to synergize the effects of such molecules.

## 5. siRNA Delivery Strategies

Even though the use of siRNAs in cancer therapy has been demonstrated as mentioned above, their chemical nature represents the main problem for the introduction in the clinical scenario. Indeed, siRNAs are characterized by a high molecular weight and a negatively charged phosphodiester backbone that impedes the crossing of biological barriers. Furthermore, their delivery to target cells is inefficient because of the presence of serum nucleases in the bloodstream [121]. Additionally, siRNAs are rapidly cleared from the blood through liver accumulation and renal filtration, which further reduce half-life [122] and cause a number of off-target effects. Moreover, siRNAs can be recognized by receptors of the innate immune system, such as the Toll-like receptors, leading to the release of pro-inflammatory cytokines [123].

Over the last decades, different siRNA-based approaches aimed at interfering with the tumoral microenvironment in breast have been developed.

Nanoparticles (NPs) and siRNA-peptide conjugates are the most used systems and allow to overcome siRNA stability and delivery problems. NPs are structures smaller than 100 nm that are used to encapsulate siRNA, improving delivery and therapeutic efficacy [121]. NPs protect the siRNAs from serum nucleases and undesirable immune responses, can carry a high quantity of RNA molecules, resist renal clearance, and their surface can be functionalized with specific ligands recognized by the target cell [124].

Hassania et al. [125] took a new path in exploring the immunotherapeutic potential of siRNA. Indeed, they developed siRNA-loaded chitosan-dextran sulfate NPs to simultaneously silence PD-1 in T cells and PD-L1 in DCs. These NPs were infused in a 4T1 breast cancer mouse model. Ex vivo analysis showed a 10-fold decrease in PD-1 and PD-L1 levels, a point at which these molecules were unable to trigger robust signaling.

As widely described above, the targeting of elements such as cytokines and growth factors that promote TME aggressiveness may represent a promising therapeutic strategy. CCL18 is a chemokine secreted by TAMs that induces EMT in different types of tumors, encouraging the formation of distal metastasis [126]. Liang et al. generated encapsulating-CCL18 siRNA nanoparticles, composed from poly(ethylene glycol)-b-poly (ε-caprolactone) (PEG-b-PCL), poly(ε-caprolactone)-b-poly (2-aminoethyl ethylene phosphate) (PCL-b-PPEEA), and PCL homopolymer, with the aim to target breast cancer TAMs. The amphiphilic PEG-b-PCL and PCL-b-PPEEA complexes arrange into nanoparticles, placing the hydrophobic PCL chains inside the core and the hydrophilic PEG and cationic PPEEA chains on the surface, where they interact with the negatively charged siRNA. Testing this type of NP at various dimensions, the group demonstrated that the largest NPs (180 nm) delivered siRNA and induced CCL18 silencing in TAMs with higher efficacy than the other sizes, leading to substantial inhibition of breast cancer cell migration [127]. The possible reason for this is that macrophages are suitable for the internalization of extraneous materials with large size [128].

In 2018, Song et al. ideated a novel dual-stage pH-sensitive NP for co-delivery of VEGF and PIGF siRNAs in order to silence their expression in TAMs and breast cancer cells [71]. VEGF and PIGF are overexpressed factors in M2-TAMs and breast cancer cells, promoting the progression and metastasis of breast cancer when released in the TME [129,130,131,132]. In detail, the NPs consisted in a cationic polyethylene glycol (PEG) with a mannose-modified trimethyl chitosan conjugate (PEG=MT) and an anionic poly-(allylamine hydrochloride)-citraconic anhydride (PAH-Cit, PC) complex. These NPs exerted robust suppression of tumor growth and lung metastasis in breast cancer-bearing mice models [71]. In the weakly acidic TME (pH 6.0–7.0), the benzamide bond between PEG and MT breaks [133,134], and the mannose and positively charged groups are exposed. This process allows not only active target uptake by TAMs overexpressing mannose receptors, but also passive target uptake by tumor cells bearing negative charges. Once inside the cells, the acidic environment of late endosome/lysosome (pH 4.5–5.5) promotes the charge-reversal of PC chains, finally inducing the release of siRNA in the cytoplasm [133,135,136].

A further promising approach used for siRNA delivery to target TME cells is bioconjugation. This consists in the formation of a complex comprising siRNAs with elements that ensure their delivery to a target site, such as biomolecules that interact with cells in specific (i.e., through receptors) or non-specific (i.e., through electrostatic charge) ways. The use of these bioconjugates improves siRNA accumulation in specific organs and target cells without the help of any transfection agents. However, these complexes have problems related to bioavailability, due to the numerous barriers to overcome, to siRNA escape from the endosome to the cytoplasm, and to their low molecular weight, which aids rapid removal from the bloodstream by renal filtration [137].

The VEGF–VEGFR axis [138] plays central roles in angiogenic processes. In order to reduce angiogenetic progression in breast cancer, Egorova et al. [70] used an L1 peptide-based polyplex for the delivery of siRNAs targeting VEGFA, VEGFR1, and endoglin transcripts in CXCR4-positive breast cancer and endothelial cells lines. Polyplexes are polymeric systems in which nucleic acids such as siRNAs are complexed with a cationic polymer through electrostatic interactions [139,140]. The chemokine SDF-1 N-terminal fragment, which binds selectively the CXCR4 receptor, was linked to a nucleic acid-binding sequence [141]. The authors demonstrated that the combination of L1-VEFGA and L1-VEGFR1 siRNA significantly reduced VEGFA and VEGFR1 expression and, consequently, restrained migration and proliferation of endothelial cells, providing a possible tool for anti-angiogenic therapy.

Another type of molecule used for siRNA delivery is aptamers. Aptamers are single-stranded nucleic acids that fold into complex structures, acquiring the ability to interact with small molecule targets with high affinity, similarly to antibodies [142,143,144,145]. Furthermore, thanks to their chemical nature, aptamers can be easily conjugated with other molecules including siRNAs [146]. An example is a conjugate composed by an aptamer for a receptor expressed on activated CD8+ T cells and an siRNA against IL-2Rα. Reduced IL-2 signaling in CD8+ T cells promotes their differentiation into memory CD8+ T cells, characterized by a long-term protective antitumor immunity [147].

## 6. Conclusions

Breast cancer remains the leading cause of cancer-related death and the most frequently diagnosed non-cutaneous malignancy in women worldwide. Most breast cancers are the result of genetic mutations originating in the epithelial cells forming the mammary gland. However, tumor development is also accompanied by changes in the surrounding environment. According to the tumor type, current treatment options include chemotherapeutics, hormonal, and antibody approaches, which render an overall survival of 5 years. Nevertheless, patients can develop tumor relapse and metastasis, and for this reason research in these fields has increased considerably. One extensively explored alternative approach is based on siRNAs: they can be highly selective and target different molecular pathways in breast cancer to influence the crosstalk with the tumor microenvironment, as described in this review. By simply customizing the sequence, a siRNA can be used to target a gene encoding a specific protein isotype and silence the gene expression of different TME cells that compose the tumor mass.

Furthermore, a cocktail of siRNAs can downregulate several pathways simultaneously in an easier manner than small molecule inhibitors or antibodies, which can only target “druggable” proteins.

Unfortunately, siRNA can have off-target effects, and for this reason the avoidance of nonspecific toxicity is a major goal in the development of siRNA-based therapy. The toxicity of siRNA is determined by the presence of complementary sequences in the transcriptome. Therefore, extensive analyses and careful preclinical tests must be performed for a given siRNA prior to use for therapeutic purposes.

The effectiveness of siRNA depends on the ability to reach the target. In a complex in vivo system, the obstacles are exacerbated by the complexity of the animal body at different levels, such as the negative charge of the cell membrane, instability in blood, poor pharmacokinetics, rapid clearance from the blood through liver accumulation and renal filtration, and Toll-like immune responses. For this reason, great effort is directed at improving delivery of siRNA.

**Figure 1:**
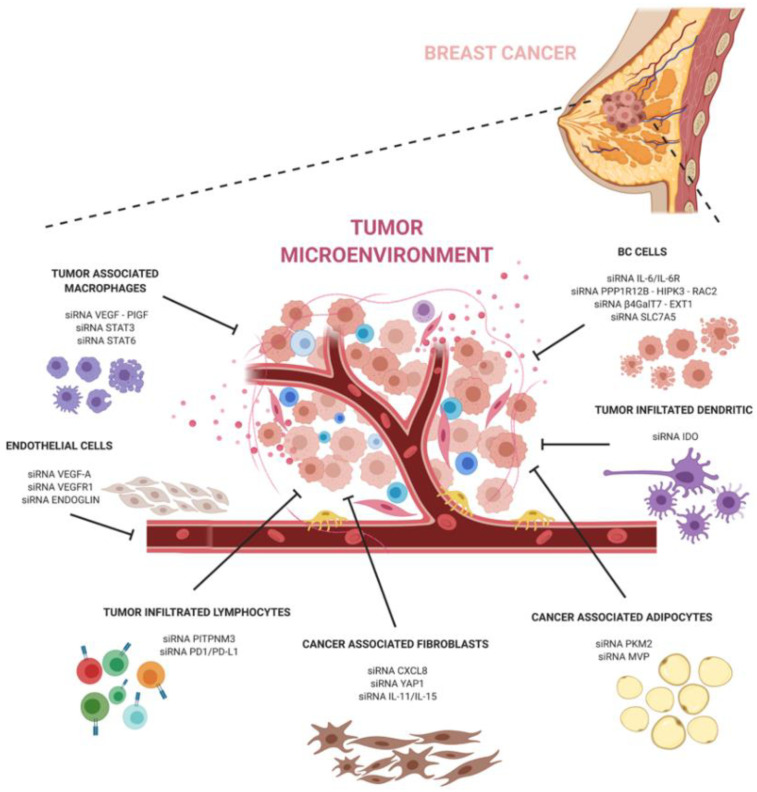
A schematic representation of siRNAs against major components of the breast cancer microenvironment. The image shows siRNAs and their targets described in the text.

The development of siRNA carriers is particularly important when targeting the tumor microenvironment. Breast cancer is known to contain abundant TME cells that can exacerbate pathways at the basis of the tumor progression, escape, and anti-tumor immune response. SiRNA-based therapeutics targeting the pro-tumoral TME can help to hinder the dangerous crosstalk between cancer and TME cells (Figure 1).

In conclusion, siRNA technology holds great promise in cancer therapy and, together with a better understanding of cancer and its microenvironment interactions, can have a great potential to revolutionize breast cancer treatment and significantly improve the outcomes of breast cancer patients.

## Figures and Tables

**Table 1:** siRNAs impairing the TME in breast cancer.

siRNA Target	TME Component	Effects	Reference Number
CXCL8	CAFs	↓ Tumor migration and invasion potentialities.	[67]
YAP1	CAFs	↓ CAF’s contraction ability and angiogenesis in vivo	[68]
IL-11, IL-15	CAFs	↓ Vascular endothelial cells’ angiogenic ability	[69]
VEGF-A VEGFR1, Endoglin	Endothelial cells	↓ Endothelial cell migration and proliferation abilities.	[69,70]
VEGF-PIGF	M2-TAMs	↓ M2-associated molecular signature, ↓ breast cancer growth and angiogenesis.	[71]
STAT6	M2-TAMs	↓ ECM remodeling and metastasis spreading.	[72]
PD1	T-cells	↓ Tumor growth in breast cancer cells bearing mice.	[73]
PKM2	CAAs	↓ EMT-related markers, inhibition of leptin-induced breast cancer cell invasion and migration in vitro.	[74]
IDO	DCs	↓ T CD4+ and Treg cells within TME.	[75]
PITPNM3	T-CD4+ cells	↓ T reg recruitment and ↑ T CD8+ populations within T cells ↑ Apoptosis in cancer cells ↓ Tumor growth and lung metastasis formation.	[76]
MVP	CAAs	↑ Accumulation of antineoplastic compounds within cancerous cells.	[77]

↑ means increase, ↓ means decrease.
